# A Comparison of Parallel Pyrosequencing and Sanger Clone-Based Sequencing and Its Impact on the Characterization of the Genetic Diversity of HIV-1

**DOI:** 10.1371/journal.pone.0026745

**Published:** 2011-10-21

**Authors:** Binhua Liang, Ma Luo, Joel Scott-Herridge, Christina Semeniuk, Mark Mendoza, Rupert Capina, Brent Sheardown, Hezhao Ji, Joshua Kimani, Blake T. Ball, Gary Van Domselaar, Morag Graham, Shane Tyler, Steven J. M. Jones, Francis A. Plummer

**Affiliations:** 1 National Microbiology Laboratory, Public Health Agency of Canada, Winnipeg, Manitoba, Canada; 2 Department of Medical Microbiology, University of Manitoba, Winnipeg, Manitoba, Canada; 3 Center for STD/HIV Research and Training, University of Nairobi, Nairobi, Kenya; 4 Genome Sciences Centre, British Columbia Cancer Research Centre, Vancouver, British Columbia, Canada; National University of Ireland Galway, Ireland

## Abstract

**Background:**

Pyrosequencing technology has the potential to rapidly sequence HIV-1 viral quasispecies without requiring the traditional approach of cloning. In this study, we investigated the utility of ultra-deep pyrosequencing to characterize genetic diversity of the HIV-1 *gag* quasispecies and assessed the possible contribution of pyrosequencing technology in studying HIV-1 biology and evolution.

**Methodology/Principal Findings:**

HIV-1 *gag* gene was amplified from 96 patients using nested PCR. The PCR products were cloned and sequenced using capillary based Sanger fluorescent dideoxy termination sequencing. The same PCR products were also directly sequenced using the 454 pyrosequencing technology. The two sequencing methods were evaluated for their ability to characterize quasispecies variation, and to reveal sites under host immune pressure for their putative functional significance. A total of 14,034 variations were identified by 454 pyrosequencing versus 3,632 variations by Sanger clone-based (SCB) sequencing. 11,050 of these variations were detected only by pyrosequencing. These undetected variations were located in the HIV-1 Gag region which is known to contain putative cytotoxic T lymphocyte (CTL) and neutralizing antibody epitopes, and sites related to virus assembly and packaging. Analysis of the positively selected sites derived by the two sequencing methods identified several differences. All of them were located within the CTL epitope regions.

**Conclusions/Significance:**

Ultra-deep pyrosequencing has proven to be a powerful tool for characterization of HIV-1 genetic diversity with enhanced sensitivity, efficiency, and accuracy. It also improved reliability of downstream evolutionary and functional analysis of HIV-1 quasispecies.

## Introduction

Cloning of PCR products and subsequent Sanger dideoxy sequencing have been widely used for the genetic analysis of HIV-1, especially in estimating the diversity of quasispecies and detecting mutations conferring antiretroviral drug resistance [1]–[4]. However, this approach is time-consuming, labor-intensive, and costly. Furthermore, polymerase induced sequence errors can confound results when sequencing cloned DNA amplified by PCR. More importantly, the number of clones that can be affordably sequenced is unlikely to adequately represent the genetic variation of the amplified viral population within a patient sample. Next-generation sequencing technology (NGS) provides the potential to greatly reduce the cost, complexity, and time required to sequence DNA without the need for cloning [5]–[7]. NGS has been applied to a broad range of applications to address diverse biological problems, including genomic sequencing, transcriptome analysis, and epigenome analysis [5], [8]. For instance, the Roche 454 pyrosequencing (454) has been used in HIV research because of its ability to provide long reads and ultra-deep coverage. Its applications include identification of rare drug resistant variants [9]–[15], prediction of HIV integration targets [16], estimation of the diversity of genital microbiota in HIV-infected women [17], and quantification of minor variants in co-receptor usage [9], [18]–[22], all of which are challenging by using Sanger clone-based sequencing.

HIV demonstrates a higher degree of genetic variation than other viruses because of the relatively low fidelity of its error-prone reverse transcriptase [23] and the high turnover rate in replication [24], [25], features which have proven to be a major obstacle to vaccine development [26]. HIV diversity is shaped by a combination of specific host-virus interactions [27]–[29], cell tropism [30], [31], immunological pressure [32]–[34], and functional constrains on viral proteins [35], [36]. Studying HIV sequence variations derived from specific patient samples can provide information on how the virus evolves and interacts with host, thus helping to develop effective strategies to control HIV infection. However, current applications of 454 in HIV research have mostly focused on identification of rare drug resistant variants and determination of cell tropism. There are not many studies exploring the potential outcome differences while profiling the HIV genetic diversity with different approaches and their consequent impacts on downstream analysis.

In this study, we compared 454 pyrosequencing with SCB in characterizing the genetic diversity of the HIV-1 *gag* quasispecies from 96 patient samples and assessed the possible contribution of pyrosequencing technology to commonly studied aspects of HIV-1 biology and evolution. We analyzed HIV-1 *gag* since HIV-1 Gag proteins are under intensive selection pressure from host immune responses, especially CTL responses, which are dominant in HIV-1 virus control in the different clade infection[37], [38]. Furthermore, the *gag* gene or proteins are also major components of HIV-1 candidate vaccines currently in clinical trials [39].

## Results

### Characterization of 454 pyrosequencing data

An average of 8,031 sequence reads per sample was produced. The generated consensus sequences of all 96 samples closely matched those from Sanger sequencing. Twenty six samples had low coverage (less than 100x) or dramatically inconsistent coverage across the target sequence and were excluded from this study. For the remaining 70 samples, 85.8% of 454 read sequences were mapped to HIV-1 *gag.* The sequence coverage varied in different regions of the HIV-1 *gag* gene, from a few hundred to over a thousand with 384 sequence reads per nucleotide position averaged over all samples (Figure S1). The details of variations at each nucleotide are shown in Table S2.

### Comparison of genetic variations generated by 454 pyrosequencing and SCB sequencing

SCB sequencing detected a total of 3,632 variations over the 1,503 nucleotides of the HIV-1 *gag* gene with an average of 53 variations per sample. By comparison, 454 pyrosequencing detected 14,034 variations at an average of 204 variations per sample (Table 1). The majority (11,050, 78.7%) of the variations detected by 454 pyrosequencing were not detected by SCB sequencing. Analysis of variation composition showed that only 33.2% (1205) of variations detected by SCB sequencing were present at an abundance of <20%. In contrast, 80.2% (11262) of variations detected by the 454 pyrosequencing were present at an abundance of <20% (Table 1). For the minor variations only detected by 454 pyrosequencing, 86% (9504) of them were present at an abundance of <10%. Within these 9504 variations, 4444 (46.8%) of them were non-synonymous mutations. Furthermore, 4215 minor variations at an abundance of <2% identified by 454 pyrosequencing were not detected by SCB.

**Table 1:** Comparison of the detected variations by 454 and Sanger Clone-based sequencing methods.

Method	Total number of variations^a^		Average number of variations/per patient	Minor variations^b^ only detected by 454 or Cloning	
	Total #^c^<20%	>20%		>10%<2%	2–10%
454 Sanger	1403411262	2772	20453	15464215	5289
	36321205	2427		642N/A	6

a : a variation is defined in this study as ‘a change’ in a nucleotide sequence (either a clonal Sanger sequence or a 454 read) compared to the consensus population-based nucleotide sequence; b: a minor variation is defined as a nucleotide with an abundance less than 20% referred to the consensus population-based nucleotide sequence; c: # represents number.

### Impact of variations in detection of gag variations on studying host virus interactions

Viral sequence variations generated by the error-prone reverse transcriptase can be selected and maintained through interactions with host immune responses as well as by functional constraints [34], [40], [41]. The ultra-deep coverage of 454 pyrosequencing can improve our understanding of viral-host interactions by reliably elucidating dominant and subdominant variations of viral quasispecies, which may not be identified by SCB sequencing. We compared the non-synonymous variations from 454 pyrosequencing to those determined by SCB sequencing in terms of their entropy scores at the known functional or immunogenic sites of Gag proteins (CTL epitopes, proteasomal cleavage sites, neutralizing Ab epitopes, Ab epitopes and assembling & packaging sites of virus), including the p17, p24, and p1p7p2p6. The difference in entropy scores from two sets of protein sequences provides a measure for the difference of composition of amino acids generated from two sequence populations (454 and SCB) that may be correlated to host immune responses. In this study, the entropy scores at the functional or immunogenic sites of the p17 and p24 generated by the two methods are significantly different (*P* = 0.0273 and *P* = 0.0302, respectively) (Table S1).

We also compared the consensus sequences derived from the two viral sequence populations in each patient and estimated the impact on studying the biology of HIV-1 Gag. We found that there is a major difference in non-synonymous variations between the two methods in 18 of 70 patients (25.7%) at 52 previously determined functional or immunogenic sites of HIV-1 Gag proteins (Table 2). Of them, 50% (26) are CTL epitopes and 34.6% (18) are neutralizing antibody epitopes. The rest (8, 15.4%) includes 4 cyclophilin A binding sites, 2 virus particle formatting sites, and 1 viral encapsulating site (Table 2).

**Table 2:** Consensus differences of HIV-1 Gag between 454 and Sanger clone-based sequencing methods overlapped with the functional of immunogenic sites in individuals.

Patient ID^a^	Clade	HLA type	Position^b^	Sequence	Function^c^
ML1111	A1	N/A	086–115	YSVHQRIDVKDTKEALEKIEEEQ**N(N/K)** KSKKK**A(T/P)**	NEUTRALIZINGVIRUSES
		N/A	121–132	DTGNS**S(S/N)**QVSQNY	NEUTRALIZING VIRUSES
		B*5703	162–169	**K(K/R)**AF**S(N/S)**PEVI	CTL RESPONSES
		B*5703	162–172	**K(K/R)**AF**S(N/S)**PEVIPMF	CTL RESPONSES
		B*4201	180–188	**T(T/I)**PQDLNTNL	CTL RESPONSES
		N/A	217–224	PV**H(Q/H)**AGPIA	CYCLOPHILIN A BINDING
ML1857	C	A*0201	077–085	SL**Y(Y/H)**N**T(T/A)**VATL	CTL RESPONSES
		B*3501	254–262	PP**I(V/I)**PVG**D(E/D)**IY	CTL RESPONSES
		A*0201	433–442	FLG**K(K/R)**IWP**S(P/S)Y(Y/H)**K	CTL RESPONSES
ML1992	A1	A*0801	074–082	E**L(L/I)**RSLYNTV	CTL RESPONSES
		A*3002	076–086	RSL**Y(F/Y)**NTVATLY	CTL RESPONSES
		A*0201	077–085	SL**Y(F/Y)**NTVATL	CTL RESPONSES
		N/A	086–115	YSVHQRIDVKDTKEALEKIEEE **Q(Q/K)**NKSKKKA	NEUTRALIZING VIRUSES
		N/A	113-122	KKAQQ**A(E/A)A(T/A)A(T/A)D(D/A)**T	NEUTRALIZING VIRUSES
		N/A	121–132	**D(D/A)**TG**H(S/N)**SS**Q(N/Q)**VSQNY	NEUTRALIZING VIRUSES
		A*0801	329–337	DC**K(K/R)**TILKAL	CTL RESPONSES
		A*0201	433–442	FLG**K(K/R)**IWPSYK	CTL RESPONSES
ML1876	A1	A*2602	028–036	KYK**L(L/M)**KH**I(I/L)**VW	CTL RESPONSES
		A*0202	077–085	SL**Y(Y/F)**NTVATL	CTL RESPONSES
		N/A	113–122	KKAQQ**A(A/E)A(A/T)A(A/T)D(A/D)**T	NEUTRALIZING VIRUSES
		N/A	121–132	**D(A/D)**TG**H(S/N)**SS**Q(Q/N)**VSQNY	NEUTRALIZING VIRUSES
ML0795	A1	A*0202	077–085	SLYNT**V(V/I)**A**T(T/V)**L	CTL RESPONSES
		N/A	392–407	CFN**C(C/Y)**GKEGHLARNC	VIRAL ENCAPSIDATION
ML1003	A1	B*5301	308–316	QASQ**E(D/E)**VK**N(N/C)**W	CTL RESPONSES
ML1102	D	N/A	064	**L(I/L)**->X	PARTICLE FORMATION
		N/A	113–121	KKAQQA**A(T/A)**ADT	NEUTRALIZING VIRUSES
		N/A	121–132	DT**G(R/G)H(N/H)**SSQVSQNY	NEUTRALIZING VIRUSES
		A*0201	433–442	FLGKIWPS**Y(H/Y)**K	CTL RESPONSES
ML1317	D	A*0201	433–442	FLGKIWPS**Y(H/Y)**K	CTL RESPONSES
ML1208	A1	N/A	064	**L(I/L)**->X	PARTICLE FORMATION
		A*0201	077–085	SLYNTVAT**L(L/I)**	CTL RESPONSES
		N/A	121–132	DTGHSS**Q(Q/K)**VSQNY	NEUTRALIZINGVIRUSES
ML1591	C	A*0201	077–085	SLYNTVA**T(T/V)**L	CTL RESPONSES
		A*0201	433–442	FLGKIWPS**Y(N/H)**K	CTL RESPONSES
ML1660	D	A*2402	028–036	KY**K(K/R)**LKHIVW	CTL RESPONSES
		N/A	086–115	YSVH**Q(Q/E)R(R/K)**I**D(E/K)V(I/V)K(K/A)** DTKEALEKIEEEQ**N(N/T)**KSKKKA	NEUTRALIZING VIRUSES
		N/A	113–122	KKAQQA**A(A/T)**ADT	NEUTRALIZINGVIRUSES
		N/A	121–132	DT**G(G/R)H(H/N)**SSQVSQNY	NEUTRALIZINGVIRUSES
ML1739	A1	N/A	113–122	KKAQQAAA**D(D/G)**T	NEUTRALIZINGVIRUSE
ML0157	A1	A*0802	180–188	TPQDLN**T(P/M)**ML	CTL RESPONSES
		N/A	217–226	PVHAQPI**A(A/P)**P	CYCLOPHILIN A BINDING
ML0415	A1	A*0301	020–028	RLRPGGKK**K(K/Q)**	CTL RESPONSES
		A*0301	020–029	RLRPGGKK**K (K/Q)** Y	CTL RESPONSES
ML0548	A1	B*57	248	**A(A/G)**->G	B*57 ESCAPING[68]
ML1594	A1	N/A	017–022	EKI**R(E/R)**LR	NEUTRALIZING VIRUSES
		B*0801	024–032	GG**K(R/K)**K**K(K/T)**Y**K(K/R)L(M/L)**K	CTL RESPONSES
		B*0801	074–082	ELRSLYN**T(T/A)**V	CTL RESPONSES
		N/A	121–132	D**T(T/A)**G**H(H/S)**S**S(S/K)Q(K/Q)**VSQNY	NEUTRALIZING VIRUSES
		N/A	217–225	PV**H(P/H)**AQP**I(V/I)**AP	CYCLOPHILIN A BINDING
ML1654	A1	N/A	121–132	D**T(A/T)**G**H(S/N)**S**S(K/S)Q(Q/K)**VSQNY	NEUTRALIZING VIRUSES
ML1768	A1	N/A	121–132	**D(A/D)**TGHSS**Q(Q/K)V(V/I)**SQNY	NEUTRALIZING VIRUSES
		N/A	217–225	PVHAGP**I(I/A**)AP	CYCLOPHILIN A BINDING

a : patient IDs are from a cohort of the Pumwani Sex Worker in Nairobi, Kenya; b: positions are referred to HXB2 *gag* gene; c: CTL epitopes are derived from the best-defined CTL epitope summary (HIV Molecular Immunology 2009, Los Alamos National Laboratory, USA). The differences between consensus sequences are shown in bold.

### Impact of the different variation detected by these two sequencing platforms on studying the evolution of the HIV-1 Gag gene

Viral sequence variation has been widely used to understand HIV-1 evolution by predicting virus-host interaction to study host immune response pressure on virus. The ability to detect major variations could influence the interpretation of host immune responses on the HIV-1. We evaluated the ability of these two methods at detecting positively selected amino acids, as one way to study HIV-1 evolution. The majority of the patients were infected by clade A1 (65.71%), with less than 35% by clade D (14.29%), C (4.29%), B (4.29%), and recombinants (11.43%). The computational method, REL [42], was used to identify the amino acid change under positive selection (PS) in HIV-1 Gag of clade A1 and to evaluate whether the results generated by pyrosequencing or SCB methods differ. Of the 42 positively selected (PS) amino acids identified in the amplified sequence populations generated by the two methods, 36 of these sites were identified by two methods. However, 6 of these sites, including amino acid 107, 163, 219, 386, 436, and 441, were only identified in sequences by 454 pyrosequencing (Table 3). These 6 sites overlap with previously identified CTL epitopes. A163G is associated with HLA class I allele B*5703 and predicted to be an escape mutation that may interrupt peptide processing of the _162_KAFSPEVIPMF_172_ epitope [43].

**Table 3:** The comparison of positively selected sites derived from 454^a^ and Sanger clone-based sequences.

*Codon*	*454 Sequences*		*Sanger clone-based Sequences*		*Difference*
	*Bayes Factor* b *PS* c		*Bayes Factor PS*		
015	177597	Yes	22717.3	Yes	No
054	2.78668e+10	Yes	2.52247e+08	Yes	No
061	20427.3	Yes	9701.84	Yes	No
062	2708.76	Yes	835.661	Yes	No
066	385327	Yes	443180	Yes	No
069	203.289	Yes	292.117	Yes	No
072	193.453	Yes	160.073	Yes	No
075	9548.06	Yes	3559.12	Yes	No
076	614542	Yes	168643	Yes	No
**107**	**173.957**	**Yes**	**69.796**	**No**	**Yes**
118	237.882	Yes	189.383	Yes	No
122	193.159	Yes	195.056	Yes	No
127	3.72494e+06	Yes	52014.3	Yes	No
143	445.954	Yes	182.668	Yes	No
146	630.075	Yes	440.031	Yes	No
147	11040.5	Yes	4416.9	Yes	No
**163**	**113.622**	**Yes**	**62.0193**	**No**	**Yes**
**219**	**105.812**	**Yes**	**9.04153**	**No**	**Yes**
223	2751.54	Yes	305.449	Yes	No
243	1.94733e+07	Yes	4.36644e+06	Yes	No
303	1.88333e+08	Yes	102877	Yes	No
310	288.617	Yes	228.149	Yes	No
315	4706.23	Yes	1870.08	Yes	No
332	1758.17	Yes	670.471	Yes	No
336	187.775	Yes	153.787	Yes	No
339	1e+25	Yes	2.43507e+10	Yes	No
370	356.917	Yes	130.182	Yes	No
372	1785.45	Yes	1049.52	Yes	No
373	105671	Yes	37326.1	Yes	No
**386**	**191.782**	**Yes**	**8.79191**	**No**	**Yes**
**436**	**106.006**	**Yes**	**43.9758**	**No**	**Yes**
**441**	**1165.93**	**Yes**	**96.7639**	**No**	**Yes**
462	1101.03	Yes	399.319	Yes	No
466	1300.13	Yes	1012.5	Yes	No
474	282428	Yes	76613.5	Yes	No
478	250.849	Yes	197.548	Yes	No
481	1628.94	Yes	872.868	Yes	No
483	224051	Yes	92560.6	Yes	No
486	35465.5	Yes	13427.5	Yes	No
487	71719.4	Yes	60727.3	Yes	No
497	3628.8	Yes	1602.01	Yes	No
498	571.563	Yes	438.724	Yes	No

Forty-six amino acid consensus sequences (subtype A1) from 454 and Sanger clone-based methods are subjected to positive selection analysis by random effect likelihood (REL) method. The result of FEL analysis is given as Bayes factor and possibility of positive selection. The differences of positively selected sites identified between two amplified sequence populations are shown in bold.

a : 454 pyrosequencing;

b : Bayes factor value (>100) is deemed as positive selection;

c : positive selection.

## Discussion

To understand the kinetics of HIV evolution in vivo, the approach being used should be able to detect the HIV quasispecies of low frequency. This is especially critical when the “minor” mutant population is of particular significance, such as in HIV drug resistance or viral escape testing. In this respect, 454 pyrosequencing technology is superior than traditional SCB method in its ability to generate a large amount of sequence data in one instrument run to achieve hundreds fold coverage at ease. Indeed, despite the large amount of data loss (67%) upon filtering for quality (from 2,360,500 to 771,011 reads) and short read length (∼102 bps) in this study, an average of 384 fold coverage was achieved to allow us to detect low abundant viral variations, especially the variations at a frequency lower than 20%, which is a challenge for SCB method. As expected, 11050 (out of 14,034) variations can only be detected by 454 pyrosequencing, which are 3 times more than the total variations identified by SCB sequencing. Moreover, the majority (80.2%) of these variations detected by 454 pyrosequencing technologies were low abundance variations (<20% in the amplified population). To achieve similar coverage using the SCB approach, at least 384 clones will need to be sequenced for each individual at a much higher cost and workload. Furthermore, the cloning procedure could introduce bias due to unintentionally selecting bigger bacterial colonies and amplification bias towards dominant quasispecies.

Previous studies have shown that 454 pyrosequencing technology can reliably detect rare variations at abundances as low as one or two percent [13], [44]–[46]. Our findings are consistent with these reports. We validated the reliability of these low abundance variations using statistical analysis based on the empirical sequencing error estimated from the experiment. We have validated 4215 variations generated by 454 pyrosequencing with frequencies of less than 2%, which is impractical for SCB methods. Thus, our study has provided additional validation of this technology in detecting low abundant variations, especially for the rare drug-resistant variations as previously reported [10], [11], [13].

Alternative methods have been developed to detect very low abundant variations (less than 1%) such as allele-specific sequencing [47], [48] and single-genome amplification (SGA)[49], [50]. Our previous study has showed that minor variations with a prevalence of 0.29% could be identified in pooled pyrosequencing [15]. In comparison with pyrosequencing, however, alternative methods are more complex and labor intensive. Moreover, allele-specific sequencing can only detect a small number of rare variations or investigate a fraction of interesting sequences [47], [51], [52]. The SGA method claims to be able to accurately represent HIV-1 quasispecies and preclude *Taq*-inducted artifacts, template switching-induced recombination, unequal template amplification, and cloning bias which are produced in Sanger cloning sequencing [49]. *Taq*-induced artifacts and template switching-induced recombination have been reported during pyrosequencing [13], [44], [45]. However, *Taq*-induced sequence errors can be considerably reduced by the described statistical method [13]. A probabilistic Bayesian approach has been developed to identify and correct PCR derived recombination errors by detecting haplotypes and estimating their frequencies using pyrosequencing data [53]. None the less, with a high sensitivity and reliability of detecting sequence variations, pyrosequencing represents a better alternative in characterizing genetic diversity of HIV-1, especially in detecting minor variations within an amplified viral population.

More importantly, this study first describes the difference in the consensus sequences (each amino acid with frequency >50%) generated by the two approaches. The consensus sequence represents the dominant sequence in an infected individual and is widely used for molecular biological and evolutionary studies of HIV-1. The magnitude of the difference between the consensus sequences generated by these two methods is big enough to affect the biological analysis of HIV-1 Gag both at population and individual levels. At the population level, we observed significant differences of the entropy scores [54] on the functional or immunogenic sites of p17 (*P* = 0.0273) and p24 (*P* = 0.0302), suggesting that there exists a significant difference in amino acid (AA) composition between two amplified sequence populations within the previously identified functional or immunogenic sites of HIV-1 Gag (Table S1). It is further supported at the individual level that the consensuses generated by these two methods are different. In 25.7% of the patients, the differences in the HIV-1 Gag consensus were found to overlap with known functional or immunogenic sites involving viral replication, assembling, packaging, CTL response, neutralizing antibody response, cyclophilin A binding (Table 2), and 50% of the amino acid differences were within CTL epitope regions [55], [56]. Thus, sequence variations between 454 pyrosequencing and SCB approaches could potentially lead to different results. Since our analyses did not include amino acid variations with frequency <50%, especially minor variations, in any given individual, the amino acid difference generated by the two methods could be underestimated. Moreover, the low abundant variations may play an important role in the functionality of HIV-1 Gag. Studies have shown that CTL epitopes with high functional avidity rapidly select for escaping mutations, resulting in low abundant variations which can be recognized by CTLs and elicit strong host immune responses [57], [58].

It is established that selection pressures exerted by host immune responses shape the genetic variation of HIV-1[27], [32], [59]–[62]. Measuring and understanding the selection pressures is an important part of evolutionary biology [34], [63], [64]. Current methods measuring selection pressures are based on protein-coding sequences. The difference in dominant variations generated by pyrosequencing and SCB methods could affect the interpretation of positive selection within amplified viral populations. Analysis using the REL method at given amino acid positions within the HIV-1 Gag proteins showed that the positive selection on those sites appears different, especially at codon 107, 163, 219, 386, 436, and 441 (Table 3). For example, for sequences generated by SCB approach, codon 163 was not identified as a positively selected amino acid; while for sequences generated by 454-pyrosequencing technologies, codon 163 was indeed identified as positively selected. Since codon 163 is within _62_KAFSPEVIPMF_172_ epitope, the difference in detecting positive selection on this site could mislead interpretation on viral interaction with the host to characterize positive selections.

Current collections of HIV sequence data in NCBI and Los Alamos HIV Sequence Database are all generated by Sanger sequencing of PCR products or clones from PCR products. These data has been widely used by research communities to study HIV-1. Our study suggests that the sequences generated by SCB method are biased towards sampling major variations in viral quasispecies population, which might result in less accurate profiling of HIV-1 genetic diversity and affect reliability of downstream functional or evolutionary analysis (i.e. positive selection). Pyrosequencing can provide much deeper sampling of the HIV-1 variations of a viral quasispecies population and a more comprehensive and accurate profiling of HIV-1 diversity. Therefore, caution should be taken in interpreting the result of SCB sequence analysis.

There was significant data loss resulting from poor quality reads and short read length of the sequences generated by pyrosequencing technology in this study. The data loss was primarily due to the early platform of sequencer, Genome Sequencer 20 (GS20), reagents and more stringent screening criteria for the high quality reads applied in this study besides its inherent shortcomings in terms of data loss during the internal 454 quality control process of reads. Despite these shortcomings, the throughput of 454 still generates datasets sufficient for this type of study. Further, the current 454 instruments (GS FLX) have substantially improved the quality (99.997% accuracy), throughput (up to 700 Mb), and read lengths (up to 700 bps). With these improvements, one can sequence the full HIV-1 genome (∼9 to 10 Kb) at 1000 fold coverage from 70 patient samples in one instrument run. However, even the best current pyrosequencing technology still cannot produce sequence reads long enough to cover the full HIV-1 genome ((∼9 to 10 kb). The assembly of pyrosequencing reads still depends on the availability of sequences generated by SCB methods.

It should be noted that there may be a limitation in using provirus instead of circulating virus in this study as not all proviral sequences produce functional virus. Even with this limitation, the use of proviral sequences in this study does not affect the comparison of 454 pyrosequencing and SCB methods, as the same starting material was used for both approaches.

In conclusion, Ultra-deep pyrosequencing has proven to be a powerful and potentially superior to the SCB method in characterizing genetic diversity of HIV-1 quasispecies, especially for the detection of low abundance variations. These consensus sequence differences observed between these methods could potentially impact the inferences made in the common studies of the function and evolution of the HIV-1 genome.

## Materials and Methods

### Subjects

The study population includes antiretroviral treatment-naïve HIV-1 positive women with chronic infections enrolled in the Pumwani Sex Worker cohort in Nairobi, Kenya. HLA class I genes had been previously typed in all subjects [43]. Both the ethics committees of the University of the Manitoba and Kenyatta National Hospital approved this study. All patients provided informed written consent for participation in this study.

### PCR amplification, cloning and sequencing with Sanger method

Proviral DNA was isolated using 1 million PBMCs from 96 HIV-1 positive women and the *gag* was amplified using nested PCR with 2 rounds of amplification. The first round PCR primers, HIV1F 5′-CTTCCCTGATTGGCAGAAY-3′ and HIV1R 5′-CAAAAATTGGGCCTGAAAATCC-3′, generates about 2,642 bps of product, and the second round primers, HIV2F 5′-AATCTCTAGCAGTGGCGCCCGAACAG-3′ and HIV2R 5′-TGGATGGCCCAAAGGTTAAACAATGG-3′, generates amplicons of about 1,997 bps. The second round PCR amplicon products were purified using the MultiscreenHTS PCR plate (Millipore Corporation) and then cloned using the TOPO TA cloning kit (Invitrogen). BigDye Terminator v3.1 (Applied Biosystems) was used to sequence *gag* with specific primers T7 5′-TAATACGACTCACTATAGGG-3′, T3 5′-ATTAACCCTCACTAAAGGGA-3′, (GSF1.6) GAGSEQF1.6 5′GATAGAGGTAAAAGACACCAAG-3′ (277-298), (GSF2) GAGSEQF2 5′-CAGCATTATCAGAAGGAGCCAC-3′ (541-562), (GOR) GAGPCRRN 5′-CTCCAATTCCCCCTATCATTTTTGGTTTCC-3′. Purified sequencing products were analyzed with an ABI 3100 Genetic Analyzer (Applied Biosystems). Nucleotide sequences were assembled and edited with Sequencher 4.8 (Genecodes Corp.). An average of 30 clones per patient was sequenced and sequences were submitted to NCBI GenBank with accession numbers GQ429817-432774**.**

### Pyrosequencing

Ultra-deep pyrosequencing was carried on the same second round PCR amplicons as used in cloning with a Roche GS20 sequencer by the Genomics Core Facility at the National Microbiology Laboratory, Public Health Agency of Canada, Canada. Briefly, the purified 96 *gag* PCR products used above were mechanically sheared, ligated to the adaptors, separately loaded into lanes (one lane/per sample) of the picolitre plate and amplified on capture beads in high-density water-in-oil emulsion picolitre reactors followed by pyrosequencing. The pyrosequencing yielded 2,360,500 sequence reads, 37.6% of which passed the default quality control, with an average read length of 102 base pairs [454 raw data was submitted to NCBI GenBank Short Read Archive (SRA) with the accession number SRA009360]. As the original PCR products may contain human or microbial DNA, non-HIV contaminant reads were filtered out by BLAST [65] alignment to the HIV-1 HXB2 reference sequence, resulting in 771,011 read sequences remaining for this study.

### Sequence alignment and determination of variations

The sequences of clones from each patient were aligned to the HIV-1 HXB2 *gag* gene by ClustalW [66]. The variations and their abundances in each sequence pool were identified by comparing the sequence of each clone to the consensus of the pool. The pyrosequencing reads from each patient were mapped onto the corresponding multiple aligned Sanger clone sequences by WUBLAST V2.0 [http://blast.wustl.edu/]. The abundance of nucleotides or amino acids at each position was calculated against the consensus sequence at each individual using Perl scripts developed in-house (available from the author on request). A variation was determined if there is ‘a change’ in a nucleotide sequence (either a clone or a read) comparing to the consensus sequence in each individual and its abundance was less than 50% in the amplified sequence population. Minor variations, defined as variations with an abundance of less than 20%, including <5% and <%1, were filtered using the reported statistical methods [13]. In this method, empirically observed distribution of error rate was applied to discriminate sequence errors from authentic variations on the assumption that the abundance of variation follows a Poisson distribution. Only variations with their abundances yielding a *P*-value of <0.001 were considered to be authentic [13].

### Measure of amino acid variability and determination of correlation between amino acid variation and HIV-1 Gag function

Entropy scores were calculated for each position in the multiple alignment for both the 454 and Sanger cloning amino acid sequences as previously described [67]. The sequence population consists of amino acid consensus sequences (one/per patient) generated by either 454 pyrosequencing or SCB method. The entropy scores from two amplified sequence populations and the paired entropy differences from the two amplified sequence populations were tested for normality using the Kolomogorov-Smirnov test. After confirmation of normality, a Student's t-test was applied to compare entropy scores (calculated from functional or immunogenic sites within HIV-1 Gag p17 and p24) of sequences generated using 454 pyrosequencing and SCB methods. The functional or immunogenic sites were defined as the amino acids overlapping previously described optimal CTL epitopes, neutralizing antibody epitopes, viral replication sites, and virus particle formation sites (HIV Molecular Immunology 2009, Los Alamos National Laboratory, USA).

### Mapping of consensus differences to HIV-1 Gag functional or immunogenic sites and positive selection analysis

The amino acid consensus differences were first determined by comparing the consensus sequences generated from each patient by the two sequencing methods and then mapped to the HIV-1 HXB2 Gag reference. The consensus differences overlapping HIV-1 Gag functional or immunogenic sites were determined. As phylogenies cannot be generated from random shotgun pyrosequencing data for quasispecies within each individual, we performed the PS analysis at the population level using the consensus sequences from each individual. RIP software (http://www.hiv.lanl.gov/content/sequence/RIP/RIP.html) was used to detect recombination between different viral subtypes of the sequences used in this study. Positive selection analysis was conducted on 46 subtype A1 consensus sequences (subtype A1) generated from the two sequencing methods using random-effects likelihood (REL) [42], [64]. The optimal time reversible substitution model was first determined for the applied sequence data and the maximum likelihood-based analysis was then carried out.

## Supporting Information

Figure S1 **The 454 read coverage at each nucleotide position of HIV-1 gag gene**. The 454 read coverage at each nucleotide position of HIV-1 gag gene was plotted for each patient. Gag nucleotide sequence positions are referred to HXB2. The average coverage of 454 reads among all the patients was highlighted in bold.
(TIF)

Table S1 **The entropy^a^ differences on functional and immunogenic sites^b^ of HIV-1 Gag proteins between 454 and Sanger cloning amino acid sequences.** a: Shannon entropy is calculated as a measure of variations in protein sequence alignments based on the method online (http://www.hiv.lanl.gov/content/sequence/ENTROPY/entropy_one.html); b: Gag protein functional and immunogenic sites are referred to HIV Sequence Databases and HIV Immunology Database (Los Alamos National Laboratory, USA); c: Paired Student *t* test was conducted. *P* value with significance is highlighted. df: degrees of freedom; CI: confidence interval.
(DOC)

Table S2 **The 454 read coverage and corresponding variation at individual level.** The 454 read coverage and its corresponding variation at each nucleotide position of HIV-1 gag gene were shown for each patient. Gag nucleotide sequence positions are referred to HXB2. p: represents patient; coverage: 454 read coverage at each nucleotide position of HIV-1 gag gene.
(XLS)
