# Supplementary material for: A Comparison of Parallel Pyrosequencing and Sanger Clone-Based Sequencing and Its Impact on the Characterization of the Genetic Diversity of HIV-1
Source: PLoS One. 2011 Oct 21;6(10):e26745. doi: 10.1371/journal.pone.0026745 (PMC3198814; doi:10.1371/journal.pone.0026745)
Supplement: Table S1 — The entropya differences on functional and immunogenic sitesb of HIV-1 Gag proteins between 454 and Sanger cloning amino acid sequences. a: Shannon entropy is calculated as a measure of variations in protein sequence alignments based on the method online (http://www.hiv.lanl.gov/content/sequence/ENTROPY/entropy_one.html); b: Gag protein functional and immunogenic sites are referred to HIV Sequence Databases and HIV Immunology Database (Los Alamos National Laboratory, USA); c: Paired Student t test was conducted. P value with significance is highlighted. df: degrees of freedom; CI: confidence interval. (DOC) [file pone.0026745.s002.doc]

**Table S1**

| *Gag* gene df Mean difference 95% CI  *P* value^c^ |
| --- |
| p17 75 0.0105 0.0012 to 0.0198 **0.0273**  p24 192 0.0071 0.0007 to 0.0136 **0.0302**  p1p7p2p6 78 0.0077 0.0040 to 0.0194 0.1932 |
